# Droplet size spectrum, velocity, angle and flow rate of pulse‐width‐modulated spray nozzles with adjuvants

**DOI:** 10.1002/ps.70932

**Published:** 2026-05-15

**Authors:** João Paulo Arantes Rodrigues da Cunha, Erdal Ozkan, Hongyoung Jeon, Heping Zhu

**Affiliations:** ^1^ Institute of Agrarian Sciences Federal University of Uberlândia Uberlândia Brazil; ^2^ Department of Food, Agricultural and Biological Engineering The Ohio State University Columbus OH USA; ^3^ Application Technology Research Unit United States Department of Agriculture Wooster OH USA

**Keywords:** agricultural spraying, atomization, pesticide application technology, PWM, surfactant

## Abstract

**BACKGROUND:**

Droplet size distribution, droplet velocity, spray angle and flow rate are key factors influencing pesticide efficacy and environmental impact. However, it remains poorly understood how these parameters are affected by the interaction among pulse‐width modulation (PWM), nozzle types and, in particular, spray adjuvants. This study investigated these factors using four flat‐fan nozzle types (standard with 80° and 110° spray angles, pre‐orifice and air‐induction). Water and two commercial adjuvant formulations [a nonionic surfactant (NIS), and a drift control agent (DCA)] were used across five PWM duty cycles (20%, 40%, 60%, 80% and 100%).

**RESULTS:**

PWM duty cycle primarily governed flow rate, which was mostly unaffected by spray solution. By contrast, droplet size distribution was strongly affected by adjuvant type, duty cycle and nozzle type. Generally, droplet size increased as duty cycle decreased for all nozzle types, regardless of spray solution. Adjuvants further modified droplet size at each duty cycle: DCA markedly increased droplet size and reduced fine droplets, whereas NIS produced a moderate increase compared with water. Both adjuvants tended to produce more uniform droplet size spectra, mitigating the PWM tendency to increase the relative span index at lower duty cycles. Decreasing the duty cycle consistently narrowed the spray angle, with the air‐induction nozzle showing the greatest sensitivity owing to incomplete sheet development under short valve‐open times.

**CONCLUSION:**

These findings demonstrate that optimizing PWM‐controlled spray performance requires not only appropriate duty cycle selection, but also the use of adjuvant formulations tailored to each nozzle type. © 2026 The Author(s). *Pest Management Science* published by John Wiley & Sons Ltd on behalf of Society of Chemical Industry.

## Graphical abstract

Optimizing PWM performance requires not only appropriate duty cycle selection, but also careful choice of adjuvant formulation tailored to each nozzle type. Drift control agents increased droplet size and reduced fine droplets, mitigating PWM‐induced variability. Decreasing the duty cycle consistently narrowed the spray angle.

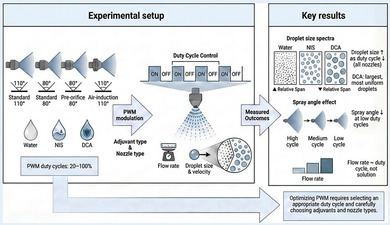

## 1 INTRODUCTION

Applying agrochemicals is essential for controlling insects, diseases and weeds to increase crop yields. Traditional hydraulic sprayers, although effective, often face challenges in maintaining consistent droplet sizes under a wide range of operating conditions. For example, changes in tractor speed during field maneuvers can cause fluctuations in operating pressure which then affect how droplets form.^1^

Pulse‐width modulation (PWM) technology has emerged as a promising solution to enhance the application precision by varying nozzle flow rates without altering system pressure. This enables real‐time adjustment of spray outputs in response to variations in travel speed, improving application uniformity and reducing environmental impact, while facilitating precise, site‐specific agrochemical delivery.^2^, ^3^, ^4^ It is currently the most widely used technique in variable‐rate spray systems worldwide.^5^

Despite its advantages, a challenge is that rapid flow rate modulations via PWM actuations could influence droplet formation dynamics, potentially affecting size distribution and velocity of droplets.^6^ Additionally, the interaction between PWM‐controlled nozzles and various spray tank mixes, including adjuvants, is not well documented. Adjuvants, such as surfactants and drift control agents, are commonly incorporated into spray solutions to modify physical properties of tank mixes such as surface tension and viscosity, which in turn can affect atomization and droplet size characteristics.^7^, ^8^, ^9^

Currently, a wide range of spray nozzle types is available on the market, each characterized by a distinct droplet formation process, thereby increasing the complexity of their interactions with PWM systems. The performance of PWM‐controlled nozzles could vary significantly by nozzle design.^6^ Standard flat‐fan nozzles produce droplets by breaking up a liquid film formed as the spray solution passes through an orifice. By contrast, pre‐orifice nozzles incorporate an internal chamber or orifice upstream of the main nozzle orifice, which reduces the exit velocity and disrupts the liquid stream, producing larger droplets and reducing drift potential.^10^ Air‐induction nozzles, however, are designed with small air inlets in the chamber upstream of relatively large outlet orifices, producing larger droplets and a smaller proportion of drift‐prone droplets compared to conventional nozzles.^11^

Given these variations, it is essential to understand the behavior of PWM‐controlled nozzles when coupled with different spray tank mixes, particularly those containing adjuvants. The interaction among nozzle type, PWM duty cycle and spray solution can influence droplet formation, thus affecting application quality.^12^ Although some studies have investigated the impact of PWM technology on droplet characteristics, most have used water as their spray solution.^6^, ^12^, ^13^, ^14^ This approach may overlook the complexities of the spray solution introduced by agrochemicals and adjuvants commonly used in agricultural applications.

Therefore, despite the increasing adoption of PWM systems, there is still limited understanding of how spray formulations, particularly adjuvants, interact with PWM duty cycles across different nozzle types. To address this knowledge gap, the objective of this research was to investigate their combined effects on droplet characteristics. Specifically, droplet velocity, droplet size distribution, spray angle and flow rate were evaluated for different flat‐fan nozzle models under varying adjuvants and PWM duty cycles.

## 2 MATERIALS AND METHODS

### 2.1 Experimental setup

The experiment was conducted under controlled laboratory conditions at USDA‐ARS ATRU co‐located in The Ohio State University – College of Food, Agricultural, and Environmental Sciences (CFAES), Wooster campus (Wooster, OH, USA). A two‐factor factorial design was used, consisting of three spray solutions and five PWM duty cycles, with three replicates per treatment for each nozzle type.

Four flat‐fan spray nozzles were evaluated, all manufactured by TeeJet Technologies (Spraying Systems Co., Wheaton, IL, USA): XR11004 and XR8004 (standard flat‐fan nozzles), AIXR11004 (air‐induction flat‐fan nozzle) and DG8004 (drift guard flat‐fan nozzle). These nozzle types were selected owing to their widespread use in agricultural spray applications.

All nozzles were expected to have a nominal flow rate of 1.58 L min^−1^ at 276 kPa in accordance with ISO 10625 standard.^15^ According to the manufacturer, the XR11004 (110° spray angle) and XR8004 (80° spray angle) are standard extended‐range flat‐fan tips that generate fine‐to‐medium droplets depending on pressure. The DG8004 (80° spray angle) features an internal pre‐orifice that produces medium‐to‐coarse droplets. The AIXR11004 (110° spray angle) employs a dual‐orifice system that entrains air into the liquid stream, producing medium‐to‐extremely coarse droplets.^16^

Three spray solutions were tested: local tap water (control) and local tap water mixed with one of two adjuvants – Preference® (nonionic surfactant) and Weather Gard Complete® (drift control agent) (Table 1). Each solution was freshly prepared before the tests to ensure adjuvants were thoroughly mixed before testing.

**Table 1:** Detailed information on spray adjuvants

Trade name	Primary ingredients	Concentration (%, v/v)	Main characteristic (abbreviation)
Preference®	Alkylphenol ethoxylate, sodium salts of soya fatty acids, isopropyl alcohol	0.5	Nonionic surfactant and antifoaming agent (NIS)
Weather Gard Complete®	Lecithin, alkylphenol ethoxylate phosphate ester, methyl soyate, dimethylpolysiloxane	0.5	Drift control agent, deposition aid, penetrant and antifoaming agent (DCA)

### 2.2 Physical properties of spray solutions

Surface tension, viscosity and density of the spray solutions were measured to investigate their potential effects on the measured parameter via their influence on spray atomization. Surface tensions were measured using a CSC Precision Tensiometer (CSC Scientific Company, Fairfax, VA, USA), according to the Du Noüy ring method.^17^ Viscosity was measured with a Viscolite 700® viscometer (Hydramotion, Malton, York, UK), which operates on the principle of resonant vibration. Density was determined by measuring the mass of 0.1 L of the solution placed in a volumetric flask, using a digital balance (XS205; Mettler Toledo, Greifensee, Switzerland) with a resolution of 0.1 mg. All measurements were conducted at a liquid temperature of 20 °C.

### 2.3 Pulse width modulation spray system

Each nozzle was electrically turned on and off using a PWM solenoid valve (55295–1‐12; Spraying Systems Co., Wheaton, IL, USA) and a spray controller (EVO Spray; Capstan AG, Topeka, KS, USA), which modulated the flow rate according to the duty cycle of the electrical signal applied to the valve. The valve operated through an internal piston mechanism actuated by electromagnetic and mechanical forces. The electromagnetic coil produced the force needed to open the valve, whereas a spring provided the restoring force to close it. A supply voltage of 14.5 VDC was provided to the controller to power the valve.

The liquid was supplied from a stainless‐steel tank through an air‐pressurized delivery system. This configuration provided a stable, constant‐pressure supply, minimizing pressure fluctuations during operation. It should be noted that this setup might differ from some pump‐driven systems used in field applications, where pressure pulsations could occur. One of the four nozzles was connected to the valve to spray continuously (100% duty cycle) at an operating pressure of 276 kPa (40 psi) using a pressure regulator. The controller operated the valve at a frequency of 10 Hz while varying the PWM duty cycle (DUC) from 20% to 100%, with a 20% increment. No further pressure adjustments were made while conducting measurements at other DUCs (20%, 40%, 60% and 80%).

The PWM frequency of 10 Hz was selected because it represents a typical operating condition commonly used in agricultural PWM systems.^18^ Additionally, the pulsing cycle at this frequency is substantially longer than the characteristic time scale of liquid sheet breakup, indicating that atomization occurs within each pulse. Commercial PWM valves generally operate within a range of 10 to 40 Hz, with 10 Hz being the most commonly used frequency because nozzle flow rates may not be modulated accurately at frequencies of >20 Hz.^19^ Thus, manufacturers mostly recommend 10 Hz to operate PWM systems for pesticide spray applications.^18^

All tests were conducted under laboratory conditions, with a mean ambient temperature of 20 °C and relative humidity (RH) of 59%.

### 2.4 Droplet size measurement

**Figure 1:**
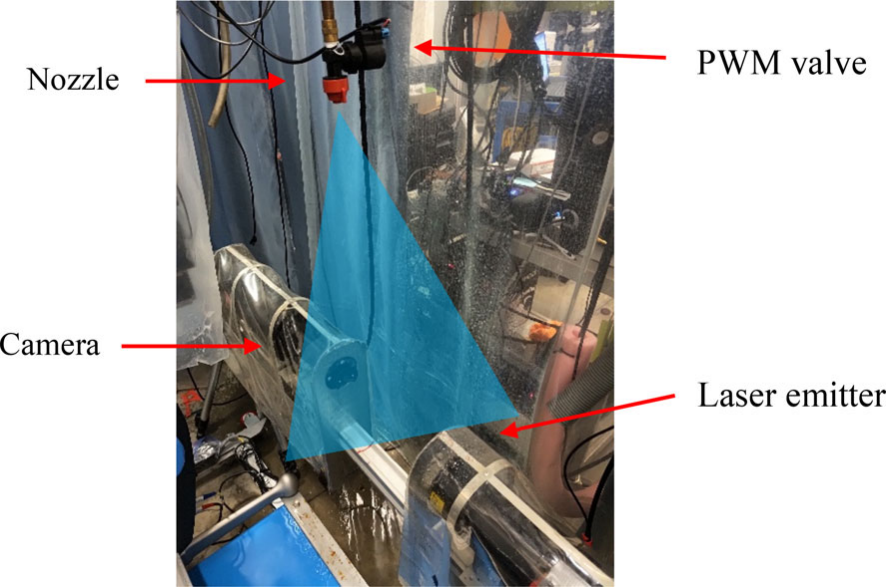
Droplet size measurement setup.

Droplet sizes of four nozzles were measured using a particle/droplet image analysis (PDIA) system (VisiSize N60; Oxford Lasers, Didcot, UK), as described previously by Wei *et al*.^13^ and Salcedo *et al*.^14^ Thus, a brief information of the measurement is provided here. The system consisted of a laser emitter, a high‐resolution digital camera and dedicated image analysis software (Fig. 1). The emitter and the camera were mounted facing each other on a rail system, with a defined measurement area located in the space between them. The nozzle, located at 0.5 m above the measurement area, discharged spray droplets travelling through the area to measure their sizes. A motorized rail system mounted above the PDIA system was used to laterally move the nozzle at a travel speed of 2 mm s^−1^ across the spray pattern to measure droplet sizes from the entire spray fan.

Once the measurement started, the laser beam from the emitter illuminated the measurement area and the camera captured droplet images at ≤30 fps. The optical system was calibrated for a pixel resolution of 7.6 μm over a 6065 × 4548 μm field of view, allowing accurate measurements of droplet diameters ranging from ≈20 to 4500 μm. For each treatment combination, the measurements were continued until ≥10 000 droplets had been recorded.

The following parameters were recorded: *D*_v10_, *D*_v50_ and *D*_v90_ (volumetric diameters at which 10%, 50% and 90%, respectively, of the spray volume comprises smaller droplets); *V*_145_ (volume percentages of droplets <145 μm); and the relative span index (a measure of droplet size uniformity), calculated as (*D*_v90_ – *D*_v10_)/*D*_v50_. The *V*_145_ parameter was selected based on the discrete size classes provided by the measurement system and was closely related to the commonly used *V*_150_ threshold (volume percentages of droplets <150 μm), widely adopted as an indicator of drift‐prone droplets. The droplet size distribution parameters (*D*_v10_, *D*_v50_, *D*_v90_ and relative span index) also are presented in Supporting information Table S1 to provide a more complete characterization of the droplet spectrum.

### 2.5 Flow rate

The nozzle flow rate was measured for each DUC–adjuvant combination as described in Wei *et al*.^13^ Therefore, a brief description of the flow rate measurement is provided here. The weight of a stainless‐steel tank containing the spray solution was continuously measured using a digital balance (resolution 1 g, IS 150 IGG‐S0CE; Sartorius Goettingen, Germany), which transmitted the weights to a computer at 10 Hz for 60 s. The recorded weight data were used to calculate the liquid flow rate for each DUC. For the corresponding liquid flow rate calculation, the density of the spray solutions was assumed to be equal to that of water at room temperature, as no significant differences were observed among the solutions (see Table 2).

**Table 2:** Surface tension, viscosity and density of the spray solutions

Spray solution	Surface tension (mN m^−1^)	Viscosity (mPa s)	Density (g cm^−3^)
Water+NIS	26.3 c	1.18 b	0.9955 a
Water+DCA	30.0 b	1.29 a	0.9957 a
Water	71.3 a	1.05 c	0.9958 a

*Note*: Values within a column followed by the same letter are not significantly different (*P* > 0.05) according to Tukey's HSD test.

Abbreviations: DCA, drift control agent; NIS, nonionic surfactant.

### 2.6 Droplet velocity measurement

**Figure 2:**
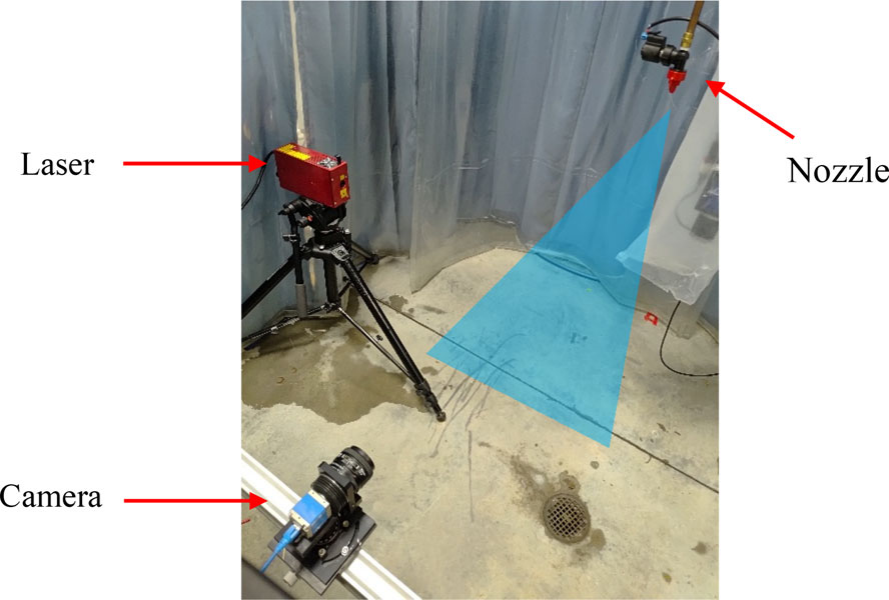
Droplet velocity measurement setup.

The effects of adjuvants and PWM operation on droplet velocity were evaluated. Droplet motion was analyzed using a particle image velocimetry (PIV) setup of an Oxford Lasers imaging system. The setup included a laser light source (Firefly 300 W; Oxford Lasers, Didcot, UK) and a digital camera (Pixelfly 270 XD; PCO, Pittsburgh, PA, USA) (Fig. 2). The camera has an image resolution of 1392 × 1024 pixels and a spatial resolution of 6.45 μm per pixel.

The laser sheet from the light source was carefully adjusted to align with the spray axis for illuminating a 10 × 10 cm region ≈50 cm below the nozzle orifice to capture droplet velocities after full atomization and breakup. After the adjustment, the laser sheet was pulsed at the time interval of 0.1 ms and the exposure of the camera was synchronized with DUC of pulsed laser sheet. While illuminating the area with a pulsed laser sheet, the camera captured two consecutive images of the same spray field.

Droplet velocity vectors were obtained using cross‐correlation algorithms embedded in the Oxford Lasers analysis software (VidPIV, Version 5; Oxford Lasers) with the time interval between consecutive frames. The mean droplet velocity for each nozzle–adjuvant–DUC combination was calculated as the magnitude of the resultant vector obtained from the horizontal (*V*_*x*_) and vertical (*V*_*y*_) velocity components, averaged >20 image pairs.

Regarding the droplet velocity evaluation for the air‐induction nozzle, we did not expect any influence of entrained air bubbles on the measurements. As shown by Guler *et al*.,^11^ droplet size is governed primarily by pressure losses within the Venturi chamber and nozzle geometry, rather than by changes in the atomization mechanism itself. In this context, any air present within the liquid stream in the nozzle chamber would be compressed and would burst at the early stage of the spray sheet formation after being discharged from the nozzle orifice.

### 2.7 Spray angle

**Figure 3:**
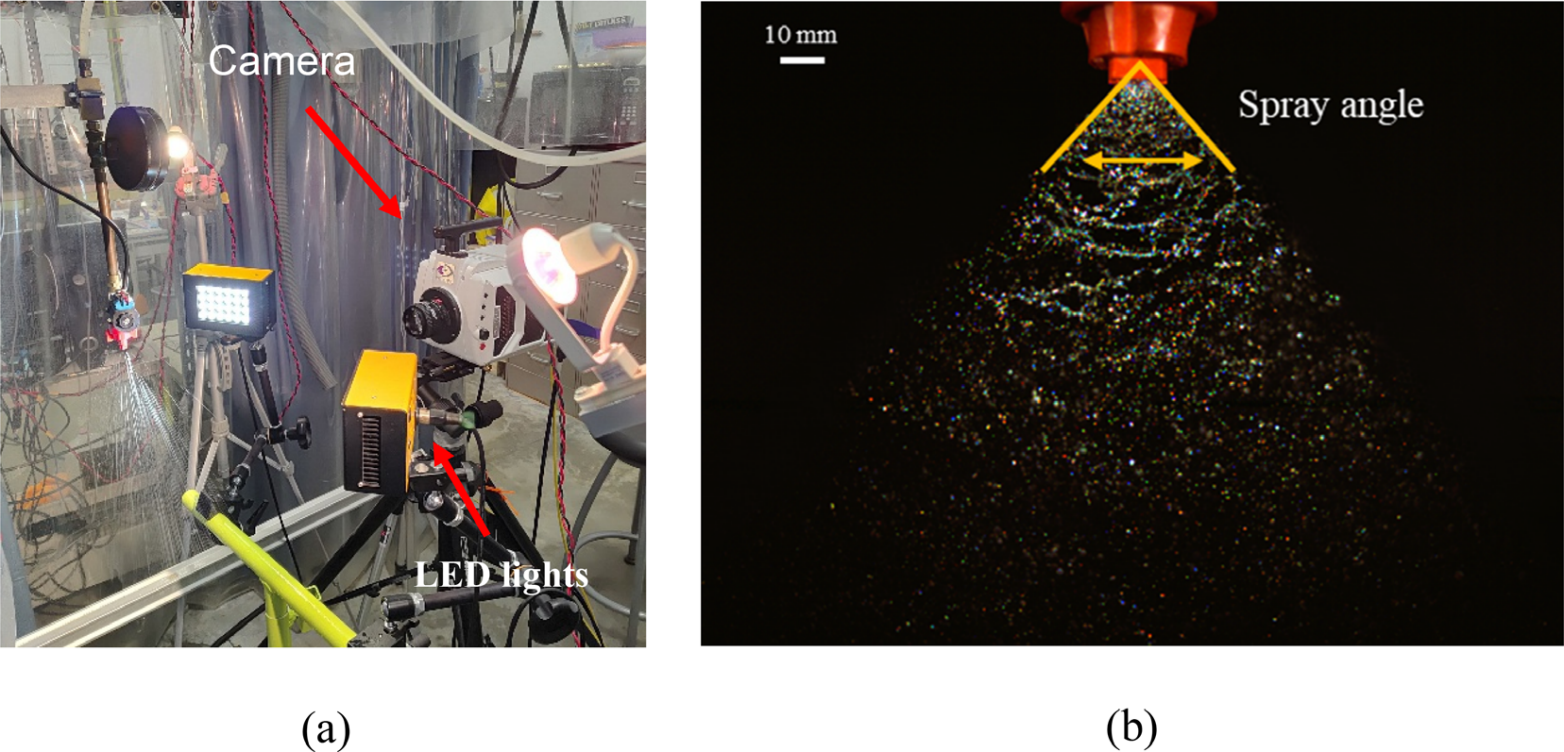
Experimental setup used for spray pattern characterization (a) and an example image used to assess the spray angle (b).

Comprehensive evaluations of the droplet formation process under different treatments are vital to understand their effects on spray characteristics. Spray fan angles served as one of the indicators for assessing spray distribution, as changes in fan angles can directly affect the area covered by the spray, the uniformity of droplet deposition, and overall application efficiency. Therefore, the spray fan angles of various treatments were measured using a high‐speed camera (Phantom v2512; Vision Research Inc., AEMTEK, Wayne, NJ, USA) and an image capturing program (Phantom Camera Control; Vision Research Inc.) [Fig. 3(a)]. The camera captured spray fan images at a resolution of 1280 × 800 pixels at 1000 fps. LED lights (Multiled LT‐V8‐11; GS Vitec, Bad Soden‐Salmunster, Germany) illuminated the spray fan area, and a black background was placed behind the nozzle to provide image contrast [Fig. 3(b)]. The camera was positioned to record the spray fan pattern during nozzle operation, thereby enabling visualization of the breakup dynamics and droplet formation across treatments.

Spray fan images captured were analyzed using image analysis software (icy, v2.5.4; Institut Pasteur, Paris, France) to determine the spray fan angle near the nozzle orifice before liquid sheet breakup. The measurements were always taken from images captured once the jet stream was fully developed.

### 2.8 Data analysis

All data from variables (flow rate, *D*_v50_, relative span index, *V*_145_, droplet velocity, and spray angle) were obtained with three replicates per treatment for each nozzle and analyzed using two‐way analysis of variance (ANOVA) to assess the effects of spray solution, DUC and their interactions. Before ANOVA, data were tested for normality of residuals using the Shapiro–Wilk test and for homogeneity of variances using Levene's test. Statistical analyses were performed using R v4.2.1^20^ at a 95% confidence level (*P* < 0.05). When significant effects were detected, mean comparisons were performed using Tukey's honestly significant difference (HSD) test for the qualitative variable (spray solution). For the quantitative variable DUC, regression models were fitted, and model selection was based on the statistical significance of the regression coefficients (*P* < 0.05), the coefficient of determination (*R* ^2^), and the choice of the simplest model that adequately described the data. For the physical properties of the spray solutions, data were analyzed using a one‐way ANOVA considering spray solution as the single factor, and means were compared using Tukey's HSD test (*P* < 0.05).

## 3 RESULTS AND DISCUSSION

### 3.1 Surface tension, viscosity and density of the spray solutions

The addition of adjuvants markedly reduced the surface tension of the spray solutions compared to water (Table 2). Among the tested tank mixes, NIS reduced the surface tension to the lowest among the three solutions, followed by DCA, whereas water exhibited the highest value. Regarding viscosity, both adjuvants increased it, with DCA showing a stronger effect. No significant differences in density were observed among the solutions. Although changing density is possible, it is difficult to achieve in practice owing to the low concentration of adjuvants relative to water.

It is important to note that DCA is a mixture of compounds, including dimethylpolysiloxane, which has a strong ability to reduce surface tension depending on its concentration.^21^ Other constituents can contribute to an increase in viscosity, such as lecithin, an amphiphilic phospholipid, which organizes into micelles, bilayers or lamellar structures in solution, forming an internal network that increases flow resistance and thereby enhances the viscosity of the spray mixture.^22^, ^23^

By contrast, NIS contains alkylphenol ethoxylate as its main component, a classic nonionic surfactant that effectively lowers surface tension but only slightly increases viscosity at higher concentrations. These findings are consistent with the known physical effects of surfactant‐type adjuvants, which primarily alter interfacial properties rather than bulk fluid resistance.^24^

The interaction between surface tension and viscosity offers insight into the mechanisms controlling droplet formation. Lower surface tension encourages liquid sheet disintegration, leading to the production of finer droplets, although this is a nozzle‐dependent process. Meanwhile, higher viscosity stabilizes the sheet and delays breakup. Consequently, the final droplet size distribution results from the balance between these two opposing forces rather than the effect of either property alone.^25^

Moreover, in PWM systems, where the spray sheet is intermittently formed and disrupted at high frequency,^13^ the relative effects of surface tension and viscosity become even more critical. Fluids with very low surface tension might produce unstable sheet formation during short pulse durations, leading to broader droplet spectra, whereas slightly more viscous formulations can promote greater stability in droplet breakup, with viscosity playing a dominant role in atomization outcomes.^26^

In the case of the drift control agent (DCA), its behavior was influenced not only by bulk viscosity, but also by its complex formulation, which might promote the formation of structured liquid phases and increased resistance to deformation during atomization. These effects could reduce the filament breakup, thereby limiting the formation of fine droplets.

### 3.2 Flow rates

**Figure 4:**
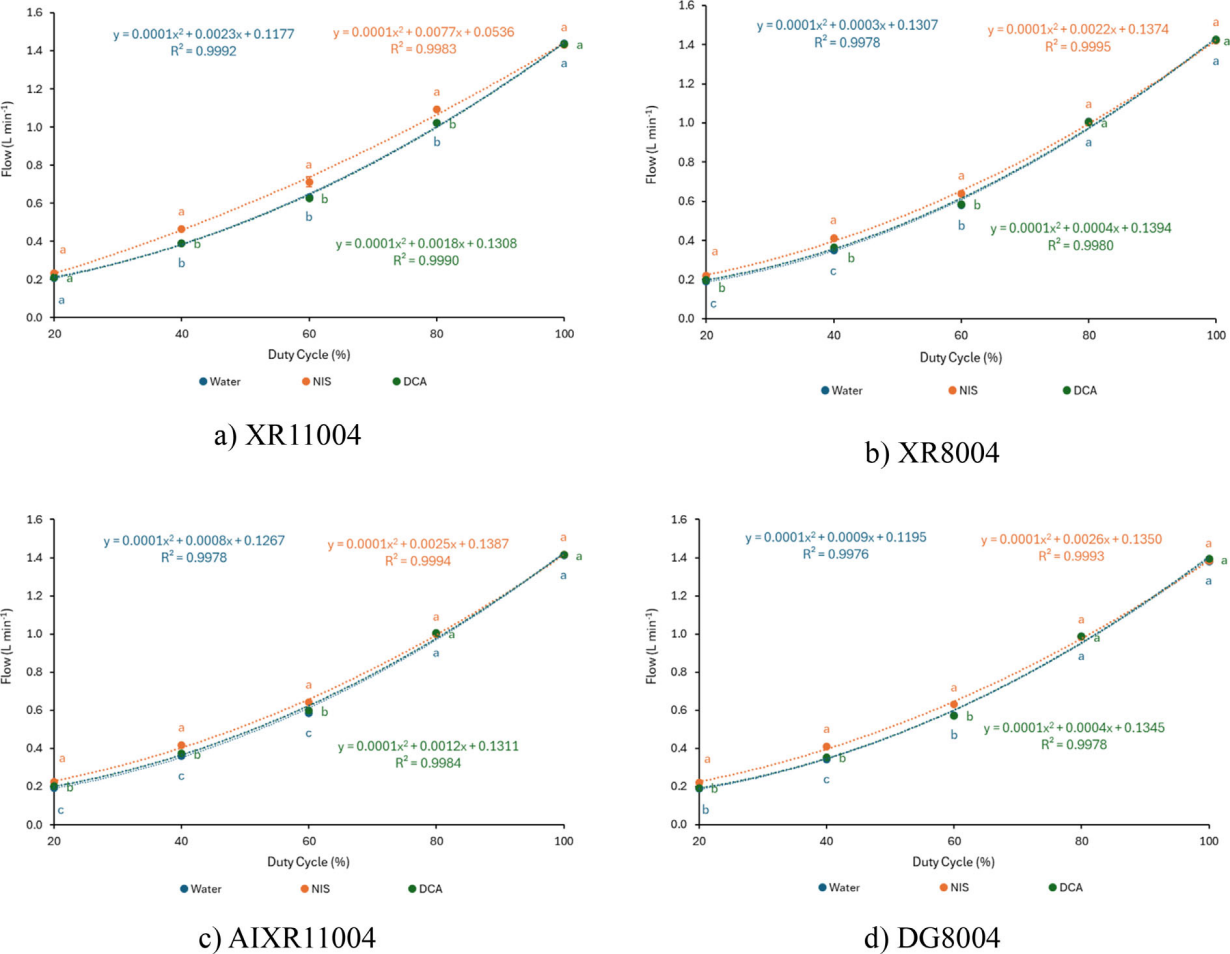
Mean flow rates of four nozzles (a) XR11004, (b) XR8004, (c) AIXR11004 and (d) DG8004 connected to a PWM solenoid valve at duty cycles from 20% to 100% (276 kPa), using different spray solutions (water, water+nonionic surfactant‐NIS, and water+drift control agent‐DCA). Points within the same duty cycle followed by the same letter are not significantly different (Tukey's HSD, *P* > 0.05). Error bars representing the SEs are invisible because they are too small.

The mean flow rate showed a quadratic increase with the PWM DUC for all four flat‐fan nozzles tested (XR11004, XR8004, DG8004 and AIXR11004), with *R* ^*2*^ > 0.99 (Fig. 4). This correlation confirmed the expected behavior of PWM‐based systems, in which the DUCs of the PWM valve are related to the effective liquid volumes discharged. The flow rate increased as the duty cycle increased from 20% to 100%, reflecting the longer valve opening time and the corresponding increase in output volume.

At equivalent DUCs, only minor differences in flow rates were observed among the spray solutions. Mixtures containing the nonionic surfactant generally produced slightly higher flow rates than water, especially at intermediate DUCs, whereas the drift control agent yielded flow rates similar to those of water. Although some of these differences were statistically significant (*P* < 0.05), they were relatively small, usually <10%, indicating that adjuvant‐induced changes in viscosity and surface tension had a limited impact on nozzle discharge under constant pressure and PWM modulation. From a practical perspective, such small variations in flow rate were unlikely to significantly affect field application rates or spray distribution. In the worst case, with the XR11004 nozzle at 60% DUC, the nonionic surfactant increased the flow rate by 12% relative to water.

At 100% DUC, flow rates were identical across all solutions because the PWM valve remained fully open. As the DUC decreased, particularly between 40% and 80%, the NIS tank mix had a tendency to increase flow rates, possibly owing to its lower surface tension, which may enhance liquid filling and valve responsiveness during shorter open intervals.

These findings suggest that the effective flow rate of PWM‐controlled nozzles is determined not only by the nominal DUC value, but also–to a lesser extent–by the dynamic response of the solenoid operation mechanism. According to Wei *et al*.,^13^ in practical terms, low DUCs may lead to small deviations from the theoretical flow predicted by the DUC.

Additionally, even at high duty cycle values (e.g. 100%), the measured flow rate might exhibit slight deviations from the nominal values reported by manufacturers owing to pressure losses within the system and the dynamic behavior of PWM components. Wei *et al*.^13^ observed that flow rates from PWM‐controlled flat‐fan nozzles could differ from theoretical predictions under various operating conditions, including at 100% duty cycle. Likewise, Fabula *et al*.^18^ demonstrated that flow rate, pressure drop and system response were influenced by the specific PWM control system, which directly affected the discharged flow. These findings support the results of the present study, indicating that small deviations from nominal flow rates were inherent to PWM‐based spray systems and should be expected under practical operating conditions.

### 3.3 Droplet sizes

**Figure 5:**
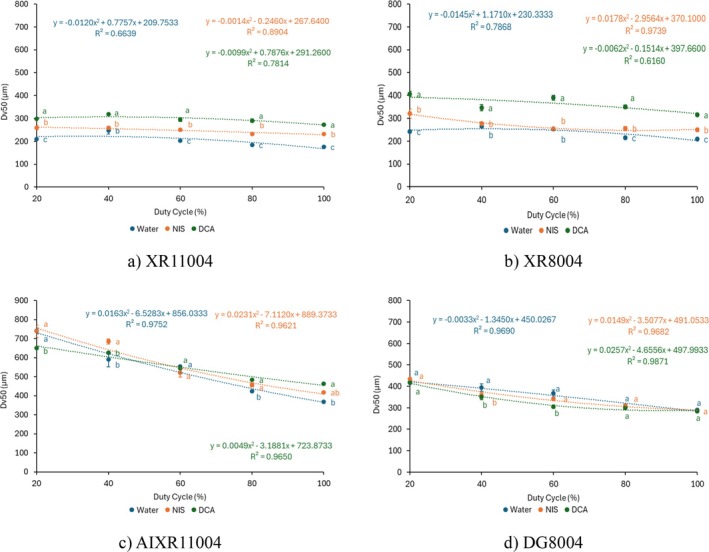
Volume median diameter (*D*_v50_) of four nozzles (a) XR11004, (b) XR8004, (c) AIXR11004 and (d) DG8004 connected to a PWM solenoid valve at duty cycles from 20% to 100% (276 kPa), using different spray solutions (water, water+nonionic surfactant‐NIS, and water+drift control agent‐DCA). Points within the same duty cycle followed by the same letter are not significantly different (Tukey's HSD, *P* > 0.05). Error bars represent the SE.

The droplet sizes (Fig. 5) showed a general reduction in the volume median diameter (*D*_v50_) with increasing DUC across all nozzle types and spray formulations, although the magnitude of this reduction varied among nozzles. This effect was more pronounced for the AIXR11004 than for the XR11004 nozzle.

Similar trends were reported by Butts *et al*.^2^ and Wei *et al*.,^13^ who, working with water, observed that lower DUCs produced coarser droplets owing to incomplete liquid sheet formation and reduced mean pressure at the nozzle, which refers to the decrease in liquid pressure at the nozzle tip caused by PWM modulation. As the DUC increased, the more continuous liquid flow enhanced sheet formation and atomization efficiency, resulting in finer droplets. This pattern was consistent across all nozzle designs.

Among the tested nozzles, as expected, the AIXR11004 (air‐induction) generated the coarsest droplets, followed by the DG8004, whereas the XR11004 and XR8004 produced finer droplets under identical operating conditions. These differences were primarily related to nozzle internal design and flow characteristics. Air‐induction nozzles entrained air and had a larger exit orifice with lower exit pressure, producing larger droplets and reducing atomization, whereas standard flat‐fan nozzles promoted greater sheet breakup and finer droplets. Pre‐orifice nozzles reduced upstream pressure, resulting in intermediate droplet sizes.

The effects of adjuvants were less consistent across nozzle types. For the XR nozzles, both adjuvants tended to increase droplet size compared to water, with the drift control agent (DCA) showing a more pronounced effect, which is likely to have resulted from its higher viscosity and polymeric composition, which helped maintain the integrity of the liquid sheet^26^ and increased resistance to ligament breakup, thereby reducing the formation of fine droplets. For DUCs in the range of 40% to 60%, particularly with the XR8004, no significant differences were observed between water and the nonionic surfactant (NIS). Their *D*_v50_ values were similar to those observed at 100% DUC, indicating that adjuvant effects observed at full DUC tended to persist at lower DUCs.

For the air‐induction nozzle, different adjuvant effects were observed at lower DUCs compared to XR nozzles. At 80% and 100% DUC, both adjuvants increased droplet size; however, at 20% DUC, the DCA produced slightly smaller *D*_v50_ values than the other treatments, whereas at 60% DUC, no differences were detected. In the case of the pre‐orifice nozzle (DG8004), adjuvant effects were less pronounced, with significant differences occurring only at 40% and 60% DUC. Overall, these findings indicate that adjuvants influenced droplet size distribution; however, this effect was clearly dependent on nozzle type,^27^ and was most pronounced for standard flat‐fan nozzles. From a practical standpoint, these changes in droplet size might influence spray drift and coverage, particularly under conditions where fine droplet fractions were critical for application efficiency.

Previous studies have shown that adjuvants can significantly alter the physical properties of spray solutions, such as surface tension and viscosity, directly affecting droplet formation and size distribution of spray nozzles. Surfactant‐based adjuvants tend to reduce surface tension, promoting the breakup of the liquid sheet into finer droplets, whereas polymer‐based adjuvants can increase viscosity and elasticity, resulting in larger droplets.^8^, ^28^ Consequently, selecting an appropriate combination of nozzle type and adjuvant is crucial to optimizing spray efficiency and minimizing drift and environmental impact.

In order to better interpret the role of fluid properties under PWM operation, it was useful to compare the characteristic timescales of pulsing and liquid sheet breakup. Nozzles controlled with 10 Hz PWM valves could have opening times from 0 to 100 ms for duty cycles from 0% to 100%. By contrast, the characteristic timescale for liquid sheet breakup in flat‐fan nozzles is on the order of milliseconds (≈2 ms), as reported in studies on liquid sheet atomization.^29^ This behavior was primarily governed by aerodynamic instabilities such as Kelvin–Helmholtz waves, which drove the primary breakup process.^30^ This indicated that sheet formation and breakup could not complete properly for atomization at low PWM duty cycles. This might be the primary reason that droplet sizes increased as duty cycles decreased. Also, surface tension and viscosity of spray solutions influenced droplet formation primarily by modifying the growth rate of instabilities and ligament formation processes, rather than interacting directly with the pulsing frequency. Consequently, the observed differences in droplet size distribution could be interpreted based on classical spray atomization mechanisms, where lower surface tension generally promoted sheet disintegration and higher viscosity delayed spray sheet breakup.

The relationship between *D*_v50_ and DUC was described using quadratic equations, primarily due to the values obtained at 20% DUC, which hindered the establishment of linear relationships. This behavior was observed previously by Gu *et al*.^31^ for adjuvant‐amended solutions and also by Butts *et al*.^2^ for the water‐only solution. For some nozzles, the model did not accurately predict *D*_v50_ across different DUCs, showing more irregular patterns, a trend likewise observed in the present work.

This increased irregularity at low duty cycles (e.g. 20%) was associated with unstable spray formation under highly intermittent flow conditions, which could lead to greater variability in droplet size measurements. From a practical perspective, such low duty cycles were generally not representative of typical field operating conditions and might lead to reduced spray uniformity. Therefore, although these results provided useful insights into the behavior of PWM systems under extreme modulation, their practical applicability is limited and should be interpreted with caution. Nevertheless, they highlighted the importance of appropriate system settings to ensure consistent spray performance.

**Figure 6:**
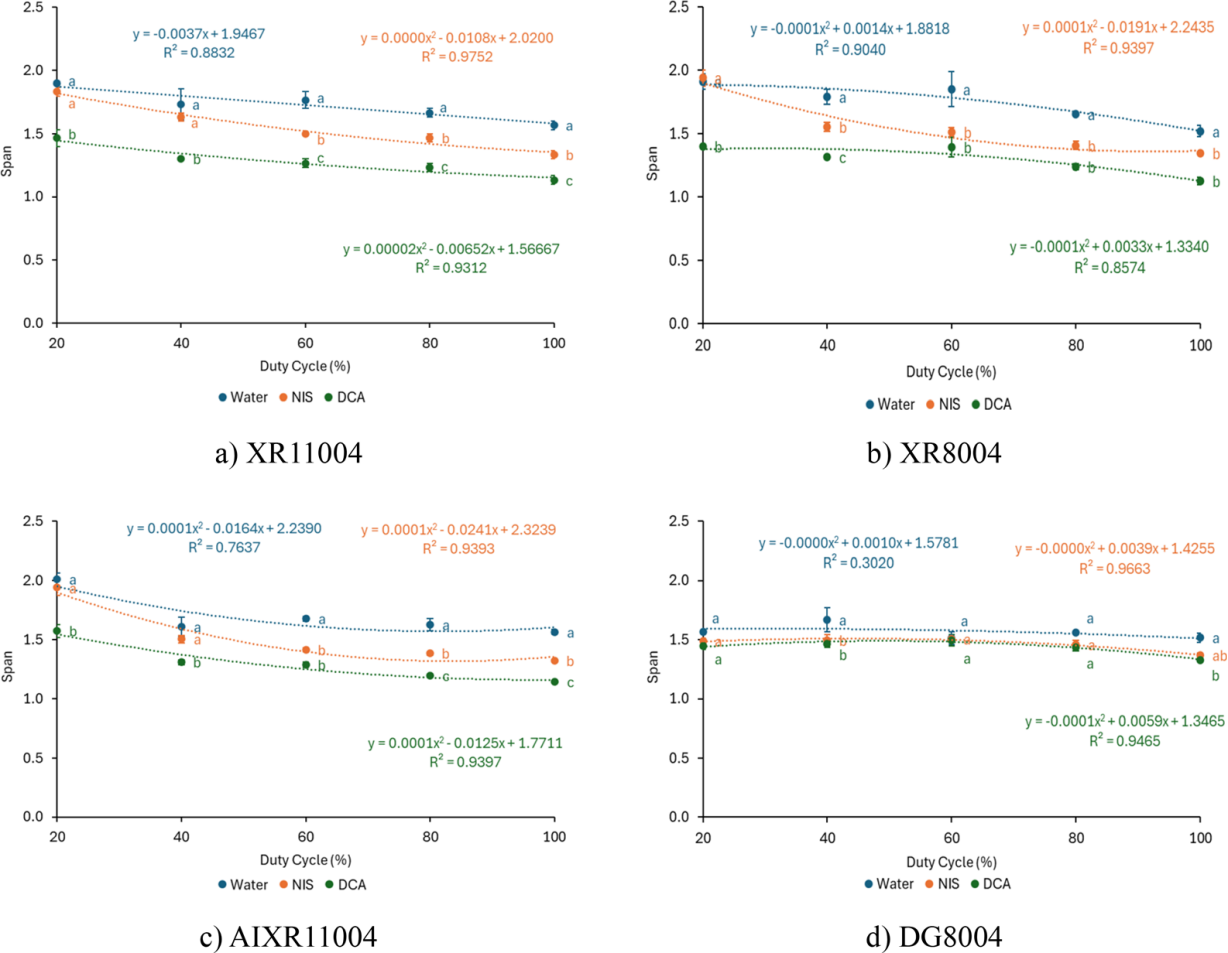
Relative span index (Span) of four nozzles (a) XR11004, (b) XR8004, (c) AIXR11004 and (d) DG8004 connected to a PWM solenoid valve at duty cycles from 20% to 100% (276 kPa), using different spray solutions (water, water+nonionic surfactant‐NIS, and water+drift control agent‐DCA). Points within the same duty cycle followed by the same letter are not significantly different (Tukey's HSD, *P* > 0.05). Error bars represent the SE.

The relative span index (Fig. 6) generally decreased with increasing DUC, indicating narrower droplet size distributions and more stable atomization at higher PWM signals. This trend was less clear for the DG8004 nozzle, which exhibited relatively stable relative span indices across DUCs. Generally, higher DUCs tended to produce more uniform droplets and a relatively closer size distribution to the distribution of 100% DUC, highlighting the strong influence of low DUCs on this relationship, which is likely to be the result of unstable sheet formation. This tendency between the indices and DUCs could be explained with quadratic relationships, as Salcedo *et al*.^14^ described.

It is evident that the addition of adjuvants in spray solutions narrowed droplet size distribution, with the effect being particularly pronounced for DCA. This finding is noteworthy, as it suggests that both adjuvants can mitigate the tendency of PWM operation to increase the relative span index, especially at low DUCs. The DCA is likely to have contributed to this behavior by increasing solution viscosity, which enhances the stability of the liquid sheet during atomization. A more viscous tank mix can damp perturbations and increase the internal cohesion of the liquid, resulting in a more stable and reproducible droplet breakup process.^32^

**Figure 7:**
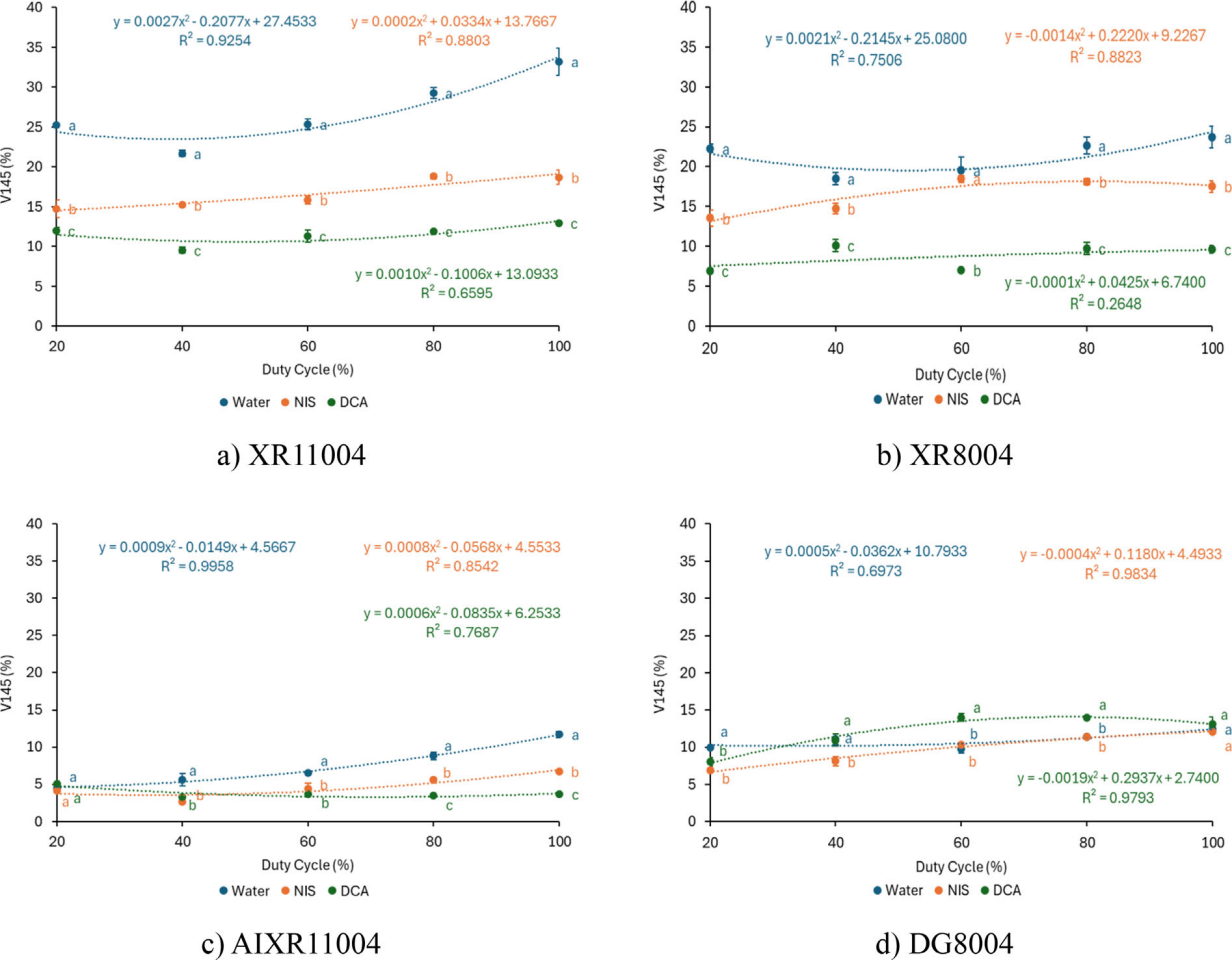
Volume percentage of droplets <145 μm (*V*_145_) of four nozzles (a) XR11004, (b) XR8004, (c) AIXR11004 and (d) DG8004 connected to a PWM solenoid valve at duty cycles from 20% to 100% (276 kPa), using different spray solutions (water, water+nonionic surfactant‐NIS and water+drift control agent‐DCA). Points within the same duty cycle followed by the same letter are not significantly different (Tukey's HSD, *P* > 0.05). Error bars represent the SE.

The *V*_145_ data (Fig. 7) confirmed the trend of increasing fine droplets as DUC increases, particularly for water sprays. For example, as *D*_v50_ decreased with increasing DUC, the volume of fine droplets tended to increase. However, this trend was less pronounced for sprays containing adjuvants. When combined with the DCA, *V*_145_ remained relatively stable across different DUCs. With few exceptions for the DG8004 nozzle, the DCA produced the lowest *V*_145_ values, highlighting its potential to reduce drift during field applications, which was particularly relevant for improving environmental safety and deposition efficiency under practical spraying conditions. No significant adjuvant effects on *V*_145_ were observed for the DG8004 at 100% DUC, and their effects were inconsistent throughout all DUCs.

These findings suggested that adjuvants might be useful for reducing the volume of drift‐prone fine droplets, particularly under lower DUCs where spray variability is higher. However, both nozzle design and solution properties must be considered to minimize drift‐susceptible fine droplets as their effect depends on the types of nozzles and adjuvants.

Overall, the results of this study were consistent with the findings of prior PWM spray research,^2^, ^13^, ^14^ confirming that droplet sizes generally increase with decreasing DUCs. This was likely because the nozzle orifice opening time decreased as the DUC decreased, thereby interfering with the droplet generation process.^13^ Although Zwertvaegher *et al*.^3^ reported contrary results, this was likely to have been attributed to higher actuation frequencies or the use of pressure‐compensating systems.

These results suggested that PWM operation and adjuvant selection could be strategically used to adjust droplet size spectra, with potential implications for optimizing spray performance and minimizing drift under field conditions.

### 3.4 Droplet velocities

**Figure 8:**
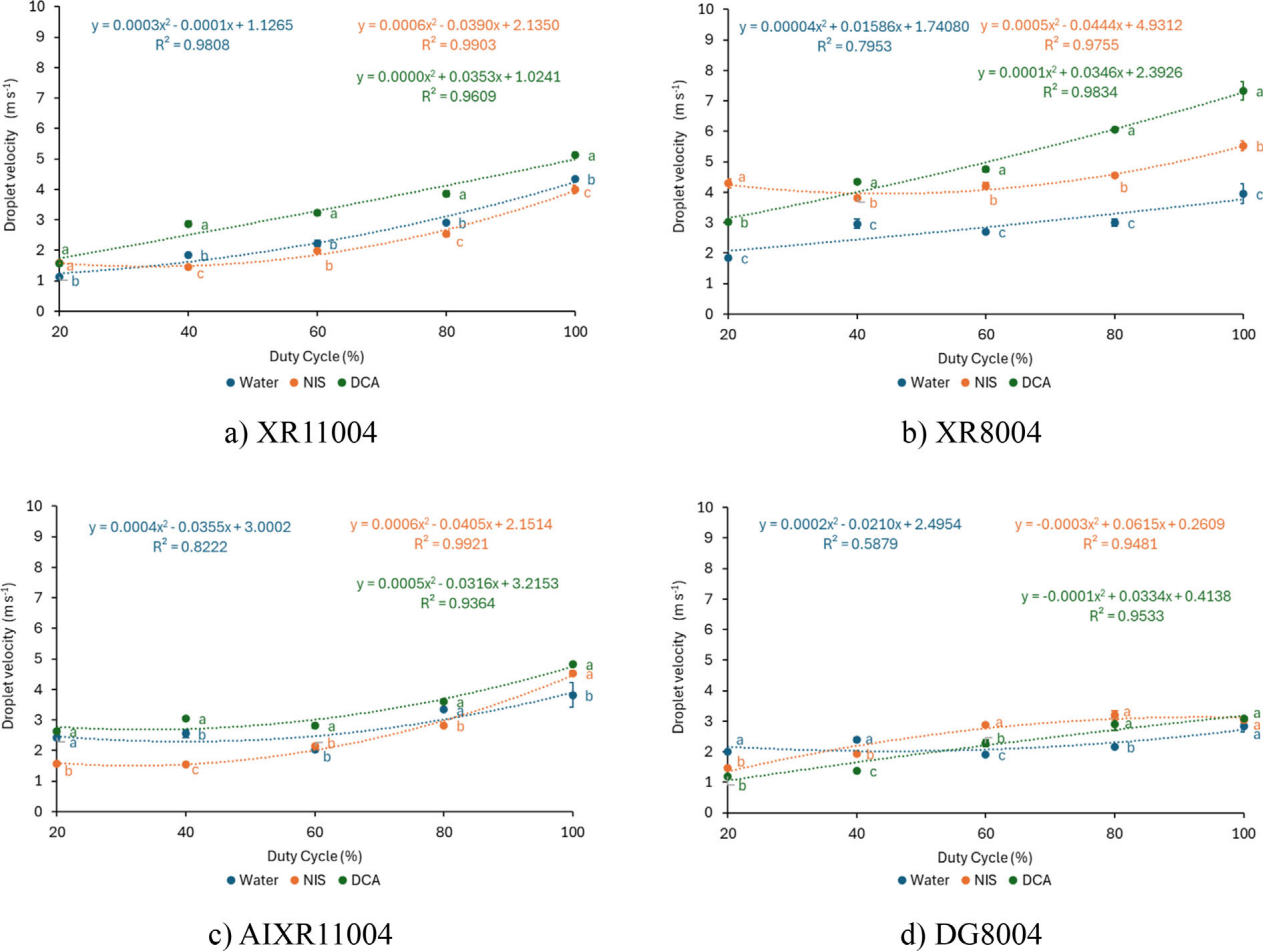
Mean droplet velocity of four nozzles (a) XR11004, (b) XR8004, (c) AIXR11004 and (d) DG8004 connected to a PWM solenoid valve at duty cycles from 20% to 100% (276 kPa), using different spray solutions (water, water+nonionic surfactant‐NIS, and water+drift control agent‐DCA). Points within the same duty cycle followed by the same letter are not significantly different (Tukey's HSD, *P* > 0.05). Error bars represent the SE.

The mean droplet velocities for all nozzles are shown in Fig. 8. The interaction between spray solution and DUC was significant across all nozzle types. In general, decreased DUC led to lower droplet velocities, with the reduction being more pronounced for standard flat‐fan nozzles, which generally exhibited higher velocities. For example, for the XR11004 nozzle, with water, the mean velocities were 4.2 m s^−1^ and 1.1 m s^−1^ when DUCs were 100% and 20%, respectively. However, this trend was not observed under all conditions. For example, for DG8004 operating with water, where the velocities were 1.9 m s^−1^ and 2.0 m s^−1^ at DUCs of 60% and 20%, respectively. As indicated by the droplet sizes, 20% DUC altered droplet‐formation behaviors differently for nozzles, preventing a reliable generalization owing to high variability in the droplet velocity data at this DUC. Similar results also were reported by Butts *et al*.^33^

The periodic interruptions of the liquid sheet in a PWM‐controlled system prevented the continuous transfer of jet energy, resulting in a loss of momentum at the end of each pulse. This pulsed flow also might induce additional turbulence, creating vortices that reduced the mean droplet velocity. As suggested by Giles and Ben‐Salem,^34^ the reduction in spray droplet velocity might be related to the effect of discharging intermittent liquid flow into the air. Because the nozzle discharged liquid into stationary air, the discharged liquid encountered air resistance to form a spray liquid fan. For intermittent spray, the spray liquid overcame air resistance after each pulse, which dampened liquid flows and increased droplet drag, consequently reducing droplet velocities. In practical terms, reduced droplet velocity might affect canopy penetration and spray deposition, particularly in dense crops. However, these results were obtained under controlled laboratory conditions, where factors such as canopy interaction, wind, and atmospheric turbulence were not considered. As a result, the direct applicability of these findings to field conditions might be limited, and further studies under real application scenarios were necessary.

Analyzing the velocity as a function of DUC for the different spray solutions revealed similar trends. The solution containing DCA resulted in higher velocities compared with the other solutions, particularly when using standard nozzles. This trend was less pronounced for air induction and pre‐orifice nozzles, which is likely to be a consequence of differences in the droplet size spectrum.

Spray solutions containing viscosity‐enhancing adjuvants tend to stabilize the liquid sheet, thereby mitigating the influence of PWM modulation on droplet velocity. Because these adjuvants also modify the droplet size spectrum (Fig. 5), and given the strong link between droplet size and velocity,^35^ it can be concluded that the spray solutions themselves affected droplet velocity.

Nuyttens *et al*.^35^ stated that droplet velocity is linked to droplet size, with larger droplets generally showing higher velocities. As observed in this study, decreasing the DUC led to an increase in droplet size. However, this increase did not result in higher droplet velocities. This was likely to have been a consequence of the effects of PWM, as the relationship between droplet size and velocity reported in the literature was not established for PWM‐controlled systems. Additionally, as mentioned previously, this system caused a pressure drop between upstream and downstream of the PWM valve, which may affect droplet velocity. A decrease in pressure may reduce the kinetic energy of discharging liquid, leading to producing larger droplets with relatively lower velocities. These results are consistent with the findings of Zwertvaegher *et al*.,^3^ who reported that increasing the pulsing frequency of a spray nozzle reduced droplet velocities, potentially increasing drift risk and decreasing canopy penetration.

It also should be noted that the present analysis considered the spray as a whole, and spatial variations within the spray fan were not explicitly evaluated. Under PWM operation, the pulsating nature of the flow might lead to localized variations in droplet size and velocity across the spray pattern, as well as temporal fluctuations within each pulse cycle. The measurements in this study represented time‐averaged values and did not resolve transient changes during the opening and closing phases of the PWM cycle. Therefore, future studies using higher spatial resolution and time‐resolved measurement techniques should be conducted to better characterize these microscale variations and their implications for nozzle arrangement and spray overlap.

### 3.5 Spray angles

**Figure 9:**
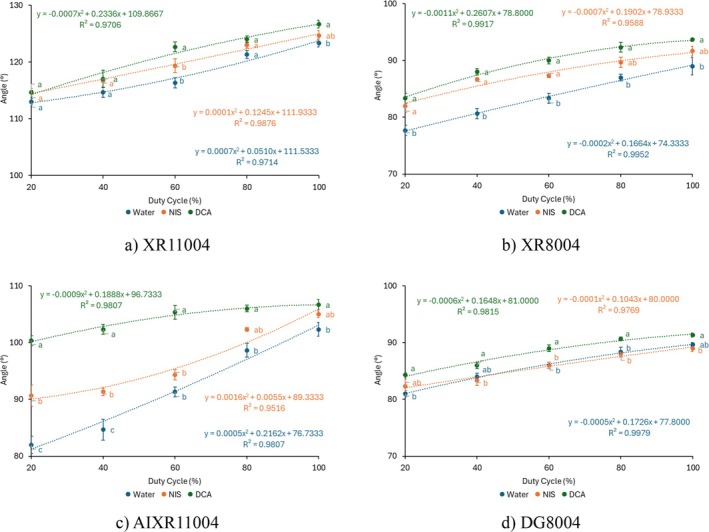
Spray angle of four nozzles (a) XR11004, (b) XR8004, (c) AIXR11004 and (d) DG8004 connected to a PWM solenoid valve at duty cycles from 20% to 100% (276 kPa), using different spray solutions (water, water+nonionic surfactant‐NIS and water+drift control agent‐DCA). Points within the same duty cycle followed by the same letter are not significantly different (Tukey's HSD, *P* > 0.05). Error bars represent the SE.

The spray angles of the four nozzle types connected to a PWM solenoid valve, measured at DUCs ranging from 20% to 100% with different spray solutions, are presented in Fig. 9. Both DUC and spray solution interacted to affect the resulting spray angle. For all nozzle types, decreasing the DUC consistently reduced the spray angle, with this effect most noticeable for the air‐induction nozzle. The spray solution also influenced the angle. At every DUC level, in general, the DCA adjuvant increased the spray angle compared to water. The NIS adjuvant also affected the angle, sometimes to a lesser extent. At 100% DUC, the effects of both adjuvants were similar across all nozzle types.

The reduction in spray angle with decreasing DUC can be attributed to the lower effective flow rates generated at shorter valve opening intervals. As DUC decreases, both pressure and flow rate were reduced, diminishing the momentum of the liquid exiting the nozzle and consequently narrowing the spray angle.^36^ This reduction could potentially influence spray coverage and uniformity, particularly at lower duty cycles. This effect was even stronger for air‐induction nozzles, which is likely to be a result of the characteristic pressure drop associated with their design.^11^

Additionally, it is important to note that the spray angles reported by manufacturers were typically determined under steady‐state conditions with continuous flow and constant pressure, which differed from the dynamic conditions imposed by PWM operation. Even under conventional (non‐PWM) conditions, studies such as Çetin *et al*.^37^ showed that measured spray angles might deviate from nominal values at a reference pressure of 276 kPa. Under PWM control, these differences can be further amplified owing to transient pressure variations and intermittent flow caused by valve actuation.

The influence of spray solution properties was consistent with findings in literature, particularly those related to surface tension. Adjuvants that alter viscosity and surface tension, such as DCA and NIS, influence the cohesion and breakup dynamics of the liquid sheet; however, Kooij *et al*.^38^ noted that surface tension remains the dominant factor in droplet formation. Hu *et al*.^39^ showed that the increase in surfactant aggregates reduces the number of intrachain interactions owing to the presence of charged chains and aggregates. As a result, the solution forms a less cohesive liquid sheet during nozzle atomization, leading to a wider spray angle. In this regard, Butler Ellis *et al*.^40^ reported that increasing the surface tension reduces the spray angle and shifts the onset of sheet oscillations, and consequently the break‐up point, farther downstream from the nozzle. For pre‐orifice nozzles, where part of the flow energy is lost inside the nozzle before atomization, the process becomes more influenced by internal geometry than by small changes in physicochemical properties of the spray solution.^41^

**Figure 10:**
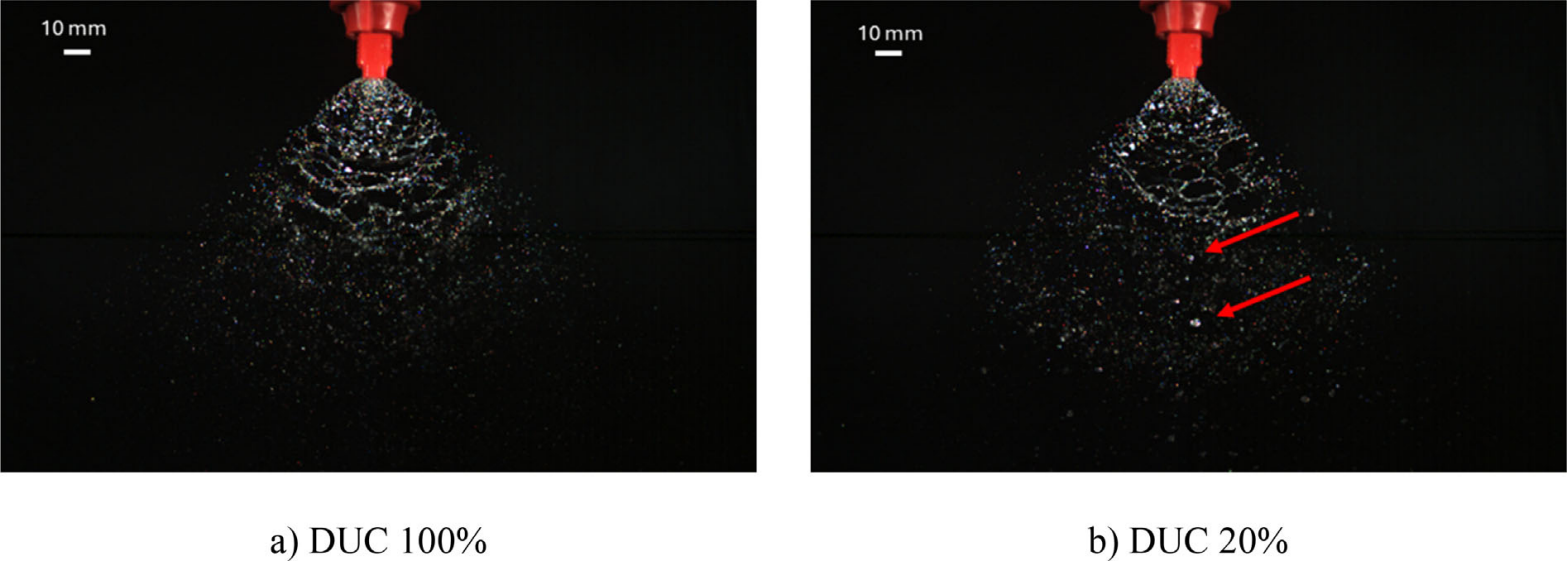
High‐speed photographs of liquid sheets produced by AIXR 11004 nozzle connected to a PWM solenoid valve, at duty cycle of 100% (a) and 20% (b) (276 kPa), using water as spray solution.

One noteworthy point observed during the image analysis for spray‐angle evaluation was the droplet‐formation behavior observed with the AIXR air‐induction nozzle. Examples of the liquid sheets produced by this nozzle operating at 20% and 100% DUC, with water as spray solution, are shown in Fig. 10. According to the general atomization mechanism of flat‐fan sprays, the liquid emerging from the nozzle initially forms a thin sheet. Surface instabilities develop as waves propagate along the sheet, leading to its fragmentation into ligaments and, subsequently, into droplets.^38^ However, depending on the spray solution, DUC, and nozzle model, this process can vary slightly, resulting in different droplet size distributions.

High‐speed images of the AIXR11004 nozzle, operated via PWM‐controlled solenoid valve at low duty cycles (20% and 40% DUC), showed incomplete spray sheet development, probably owing to insufficient fluid energy. This behavior is consistent with the substantial pressure drop characteristic of air‐induction nozzles, which occurs between the spray boom and the nozzle orifice owing to internal flow restriction and the Venturi air‐induction mechanism.^11^

This resulted in droplet size distributions distinct from those at 100% DUC. The spray exhibited a narrower and more irregular fan pattern, characterized by visible pulses of liquid discharge and a heterogeneous droplet spectrum [red arrows in Fig. 10(b)]. This issue also occurred with the adjuvants. By contrast, the conventional flat‐fan nozzles (without air‐induction) exhibited smoother and more consistent droplet formation, with no visible irregularities in flow or breakup behavior.

It is worth noting that this deformation of the jet stream was not reflected in the droplet size measurements obtained with the VisiSize N60 system used in this study, because the instrument automatically excludes nonatomized or irregular jet segments from the analysis. In particular, the shape‐rejection function in the visisize software was enabled to remove overlapping or irregularly shaped droplets.

The experiments were conducted under constant‐pressure conditions, which may differ from some pump‐driven field systems where pressure pulsations can occur. Such fluctuations may increase variability in spray characteristics, particularly at low duty cycles. However, the main trends observed in this study, including the effects of duty cycle, nozzle type and spray solution on flow rate, droplet size and velocity, are governed by atomization mechanisms and are expected to remain valid under field conditions, although potentially with greater variability.

It also should be noted that only two commercial adjuvant types (NIS and DCA) were evaluated, which may not represent the full range of commercially available formulations. Further studies including a wider variety of adjuvants are needed to validate the generality of these findings.

## 4 CONCLUSIONS

Flow rate was primarily controlled by the PWM DUC and remained mostly consistent across different spray solutions. Minor deviations observed for low‐surface‐tension mixtures, such as NIS, were generally small (<10%) and occurred at DUCs of <80%. These findings demonstrate that PWM provides reliable control of liquid delivery, whereas droplet characteristics are governed by interactions between adjuvants and DUC during PWM‐driven atomization.

Decreasing DUC generally increased droplet size across all nozzle types, whereas adjuvants further influenced droplet characteristics at each DUC, with DCA markedly increasing droplet size and reducing fine droplets, and NIS slightly increasing droplet size compared to water. The use of adjuvants produced more uniform droplets by narrowing the droplet size distribution, mitigating the PWM‐induced increase in relative span at lower DUCs. These results reveal that adjuvants play a key role in modifying atomization behavior under PWM, with effects dependent on nozzle design.

Decreasing the DUC generally reduced droplet velocity, especially with standard flat‐fan nozzles. At very low DUC (20%), this effect is inconsistent owing to high variability and pulsed flow dynamics. Viscosity‐enhancing adjuvants can partially increase velocity by stabilizing the liquid sheet, but droplet size alone does not determine velocity under PWM.

Decreasing the DUC consistently narrowed the spray angle, with air‐induction nozzles showing the greatest sensitivity owing to incomplete sheet development at short valve‐open times. Adjuvants, especially those that reduce surface tension, increased the spray angle across all DUC levels. High‐speed imaging showed that AIXR11004 air‐induction nozzle exhibited irregular atomization at low DUC, whereas conventional flat‐fan nozzles maintained more stable spray formation, highlighting differences in atomization mechanisms under PWM.

Overall, this research demonstrates that PWM spray performance is governed not only by duty‐cycle adjustment, but also by the interaction between adjuvants and nozzle type, which directly affects atomization dynamics. These findings provide new insights into PWM‐driven atomization, and support the more effective selection of adjuvants and operating parameters to improve spray uniformity, reduce drift risk and enhance the environmental sustainability of agricultural spraying.

## CONFLICT OF INTEREST

All authors declare no conflicts of interest.

## Supporting information


**Table S1.** Droplet size distribution parameters (*D*_v10_, *D*_v50_, *D*_v90_ and relative span index) for different nozzles, spray solutions and duty cycles (276 kPa).

## Data Availability

The data that support the findings of this study are available from the corresponding author upon reasonable request.
