# Supplementary material for: Droplet size spectrum, velocity, angle and flow rate of pulse‐width‐modulated spray nozzles with adjuvants
Source: Pest Manag Sci. 2026 May 15;82(9):8831–43. doi: 10.1002/ps.70932 (PMC13453229; doi:10.1002/ps.70932)
Supplement: Supplementary file 1 [file PS-82-8831-s001.docx]

# Table S1. Droplet size distribution parameters (D_v10_, D_v50_, D_v90_, and relative span index) for different nozzles, spray solutions, and duty cycles (276 kPa).

| Nozzle | Spray solution^a^ | DUC (%) | D_v10_ (µm) | D_v50_ (µm) | D_v90_ (µm) | Span |
| --- | --- | --- | --- | --- | --- | --- |
| AIXR11004 | DCA | 20 | 190 | 652 | 1214 | 1.57 |
| AIXR11004 | DCA | 40 | 215 | 627 | 1035 | 1.31 |
| AIXR11004 | DCA | 60 | 203 | 546 | 906 | 1.29 |
| AIXR11004 | DCA | 80 | 202 | 484 | 780 | 1.19 |
| AIXR11004 | DCA | 100 | 195 | 463 | 726 | 1.15 |
| AIXR11004 | NIS | 20 | 207 | 739 | 1641 | 1.94 |
| AIXR11004 | NIS | 40 | 238 | 685 | 1269 | 1.50 |
| AIXR11004 | NIS | 60 | 188 | 521 | 924 | 1.41 |
| AIXR11004 | NIS | 80 | 171 | 458 | 804 | 1.38 |
| AIXR11004 | NIS | 100 | 162 | 419 | 715 | 1.32 |
| AIXR11004 | Water | 20 | 208 | 741 | 1698 | 2.01 |
| AIXR11004 | Water | 40 | 181 | 592 | 1126 | 1.61 |
| AIXR11004 | Water | 60 | 167 | 555 | 1098 | 1.68 |
| AIXR11004 | Water | 80 | 151 | 424 | 841 | 1.63 |
| AIXR11004 | Water | 100 | 138 | 367 | 713 | 1.57 |
| DG8004 | DCA | 20 | 155 | 417 | 760 | 1.45 |
| DG8004 | DCA | 40 | 141 | 351 | 654 | 1.46 |
| DG8004 | DCA | 60 | 128 | 305 | 583 | 1.50 |
| DG8004 | DCA | 80 | 128 | 299 | 558 | 1.44 |
| DG8004 | DCA | 100 | 131 | 285 | 510 | 1.33 |
| DG8004 | NIS | 20 | 161 | 434 | 808 | 1.49 |
| DG8004 | NIS | 40 | 154 | 359 | 690 | 1.49 |
| DG8004 | NIS | 60 | 143 | 342 | 658 | 1.51 |
| DG8004 | NIS | 80 | 139 | 311 | 593 | 1.46 |
| DG8004 | NIS | 100 | 136 | 285 | 526 | 1.37 |
| DG8004 | Water | 20 | 145 | 419 | 800 | 1.56 |
| DG8004 | Water | 40 | 143 | 394 | 799 | 1.67 |
| DG8004 | Water | 60 | 146 | 367 | 701 | 1.51 |
| DG8004 | Water | 80 | 139 | 306 | 616 | 1.56 |
| DG8004 | Water | 100 | 134 | 289 | 572 | 1.52 |
| XR11004 | DCA | 20 | 138 | 298 | 571 | 1.47 |
| XR11004 | DCA | 40 | 147 | 319 | 561 | 1.30 |
| XR11004 | DCA | 60 | 140 | 295 | 510 | 1.27 |
| XR11004 | DCA | 80 | 138 | 290 | 500 | 1.23 |
| XR11004 | DCA | 100 | 134 | 273 | 446 | 1.13 |
| XR11004 | NIS | 20 | 130 | 260 | 609 | 1.83 |
| XR11004 | NIS | 40 | 128 | 260 | 553 | 1.63 |
| XR11004 | NIS | 60 | 126 | 250 | 496 | 1.50 |
| XR11004 | NIS | 80 | 118 | 232 | 455 | 1.47 |
| XR11004 | NIS | 100 | 119 | 232 | 434 | 1.33 |
| XR11004 | Water | 20 | 103 | 210 | 504 | 1.90 |
| XR11004 | Water | 40 | 109 | 247 | 540 | 1.73 |
| XR11004 | Water | 60 | 102 | 203 | 461 | 1.77 |
| XR11004 | Water | 80 | 97 | 184 | 400 | 1.67 |
| XR11004 | Water | 100 | 93 | 175 | 370 | 1.57 |
| XR8004 | DCA | 20 | 161 | 406 | 729 | 1.40 |
| XR8004 | DCA | 40 | 145 | 346 | 602 | 1.32 |
| XR8004 | DCA | 60 | 163 | 390 | 706 | 1.40 |
| XR8004 | DCA | 80 | 147 | 350 | 580 | 1.24 |
| XR8004 | DCA | 100 | 147 | 315 | 502 | 1.13 |
| XR8004 | NIS | 20 | 132 | 320 | 755 | 1.94 |
| XR8004 | NIS | 40 | 129 | 277 | 559 | 1.55 |
| XR8004 | NIS | 60 | 120 | 253 | 504 | 1.51 |
| XR8004 | NIS | 80 | 120 | 255 | 479 | 1.41 |
| XR8004 | NIS | 100 | 121 | 249 | 455 | 1.34 |
| XR8004 | Water | 20 | 107 | 242 | 570 | 1.91 |
| XR8004 | Water | 40 | 115 | 264 | 589 | 1.79 |
| XR8004 | Water | 60 | 112 | 253 | 578 | 1.85 |
| XR8004 | Water | 80 | 105 | 215 | 461 | 1.66 |
| XR8004 | Water | 100 | 103 | 210 | 422 | 1.52 |

Values represent means of three replicates.

^a^ DCA: water + drift control agent; NIS: water + nonionic surfactant.
